# lncRNA CASC19 Contributes to Radioresistance of Nasopharyngeal Carcinoma by Promoting Autophagy via AMPK-mTOR Pathway

**DOI:** 10.3390/ijms22031407

**Published:** 2021-01-30

**Authors:** Hongxia Liu, Wang Zheng, Qianping Chen, Yuchuan Zhou, Yan Pan, Jianghong Zhang, Yang Bai, Chunlin Shao

**Affiliations:** Institute of Radiation Medicine, Shanghai Medical College, Fudan University, Shanghai 200032, China; 18111140003@fudan.edu.cn (H.L.); 19111140004@fudan.edu.cn (W.Z.); 17111140001@fudan.edu.cn (Q.C.); 20111140003@fudan.edu.cn (Y.Z.); swallowpan@fudan.edu.cn (Y.P.); zjh551268@fudan.edu.cn (J.Z.); yangbai@fudan.edu.cn (Y.B.)

**Keywords:** lncRNA CASC19, radioresistance, autophagy, AMPK/mTOR, nasopharyngeal carcinoma

## Abstract

Nasopharyngeal carcinoma (NPC) is one of the most frequent head and neck malignant tumors and is majorly treated by radiotherapy. However, radiation resistance remains a serious obstacle to the successful treatment of NPC. The aim of this study was to discover the underlying mechanism of radioresistance and to elucidate novel genes that may play important roles in the regulation of NPC radiosensitivity. By using RNA-seq analysis of NPC cell line CNE2 and its radioresistant cell line CNE2R, lncRNA CASC19 was screened out as a candidate radioresistance marker. Both in vitro and in vivo data demonstrated that a high expression level of CASC19 was positively correlated with the radioresistance of NPC, and the radiosensitivity of NPC cells was considerably enhanced by knockdown of CASC19. The incidence of autophagy was enhanced in CNE2R in comparison with CNE2 and another NPC cell line HONE1, and silencing autophagy with LC3 siRNA (siLC3) sensitized NPC cells to irradiation. Furthermore, CASC19 siRNA (siCASC19) suppressed cellular autophagy by inhibiting the AMPK/mTOR pathway and promoted apoptosis through the PARP1 pathway. Our results revealed for the first time that lncRNA CASC19 contributed to the radioresistance of NPC by regulating autophagy. In significance, CASC19 might be a potential molecular biomarker and a new therapeutic target in NPC.

## 1. Introduction

Nasopharyngeal carcinoma (NPC) is an epithelial carcinoma arising from the nasopharyngeal mucosal lining. Although the new cases of nasopharyngeal carcinoma only accounts for 0.7% of all cancers diagnosed in 2018, it is notably associated with an extremely unique pattern of unbalanced geographical distribution features, that is, more than 70% of new cases occur in East Asia and Southeast Asia [1,2]. Owing to the anatomical characteristics and the relative sensitivity to ionizing radiation (IR), radiotherapy is the primary treatment option for NPC patients. Nevertheless, the local recurrence and distant metastasis are still main obstacles for the long-term survival of NPC patients due to radioresistance [3]. Hence, it is necessary to explore novel molecular mechanisms and therapeutic targets to counter the radioresistance of NPC.

Long noncoding RNAs (lncRNAs) are a group of highly conserved transcripts comprising more than 200 nucleotides in length without protein-coding potential [4]. It is well established that lncRNAs, acting as tumor suppressors or oncogenes, are tightly associated with the progression and development of many types of tumors including NPC [5,6,7,8,9]. Moreover, accumulating evidence has demonstrated that lncRNAs are involved in the regulation of tumor radioresistance via transcriptional, posttranscriptional, and epigenetic modifications. For example, lncRNA DNM3OS could bind to transcription factor FOXO1, resulting in the increase in radioresistance through the PDGFβ/PDGFRβ/FOXO1 signaling pathway [10]. Recently, the ceRNA regulatory network analysis has been proposed as one kind of posttranscriptional regulation mechanism. It was reported that LINC00963 promoted tumorigenesis and radioresistance in breast cancer by sponging miR-324-3p and inducing ACK1 expression [11]. In addition, as an important epigenetic regulator, lncRNAs may act as a scaffold modulating DNA repair in response to damage [12] and can be involved in the radioresistance [13]. For instance, radiation-induced lncRNA PARTICLE influenced the outcome of radiotherapy by forming a DNA-lncRNA triplex at a CpG island upstream of the MAT2A promoter and serving as the scaffold for MAT2A [14,15]. Additionally, lncRNAs participated in the regulation of the radiosensitivity of NPC as well. It was found that lncRNA of plasmacytoma variant translocation 1 (PVT1) was a potential target to improve the efficacy of radiotherapy for NPCs by regulating DNA repair and cell apoptosis [7]. Those studies suggested that lncRNAs might serve as new potential molecular targets for radiosensitivity.

CASC19 is a newly discovered lncRNA and locates on the 8q24 region of chromosome [16]. This lncRNA has been confirmed to be associated with the deterioration of human tumors of colorectal cancer [17] and gastric cancer [18]. Evidence suggests that the overexpression of CASC19 in Clear Cell Renal Cell Carcinoma (ccRCC) might play an oncogenic role in cancer progression [19], and knockdown of CASC19 suppressed cell proliferation, migration, and invasion in nonsmall cell lung carcinoma (NSCLC) [20,21]. In particular, CASC19 was significantly regulated in colon cancer cell lines after the treatment with different chemotherapeutic drugs [22]. However, the functional roles of CASC19 and its underlying mechanisms in the radiosensitivity of NPC have rarely been reported.

Radiotherapy, as a powerful tool to restrain tumor growth, can induce autophagy in some cancers. Autophagy is also a crucial mechanism in the regulation of radiosensitivity in addition to DNA damage repair, cell cycle arrest, apoptosis, cancer stem cells regulation, and the epithelial–mesenchymal transition [23]. Under some status, autophagy may enhance radiosensitivity by inducing impaired cell death [24,25]. However, in other cases, autophagy may contribute to radioresistance by protecting cells rather than causing cell death in various tumors [26,27,28]. At present, early-stage clinical studies are paving the way to bring forward autophagy inhibition as a radiosensitizing treatment to cancer patients, including the combination treatment of conventional radiochemotherapy (IR + temozolomide) and autophagy inhibitor (chloroquine) in glioblastoma multiforme (NCT02378532, NCT02432417) [29]. As for the role of autophagy in radiosensitivity, still remaining controversial, it is worth investigating the role of autophagy in the regulation of the radiosensitivity.

Until now, the relationship between lncRNAs, autophagy, and the radioresistance of NPCs is very rare in the literature. In the study, gene sequencing was employed to comprehensively and systematically screen the candidates of lncRNAs associated with radioresistance using our previously established radioresistant NPC cell line (CNE2R) [30]. It was identified that lncRNA CASC19 could sensitize NPC cells to radiation by blocking autophagy and promoting apoptosis via the AMPK-mTOR pathway.

## 2. Results

### 2.1. Validation of Radioresistant NPC Cells

**Figure 1:**
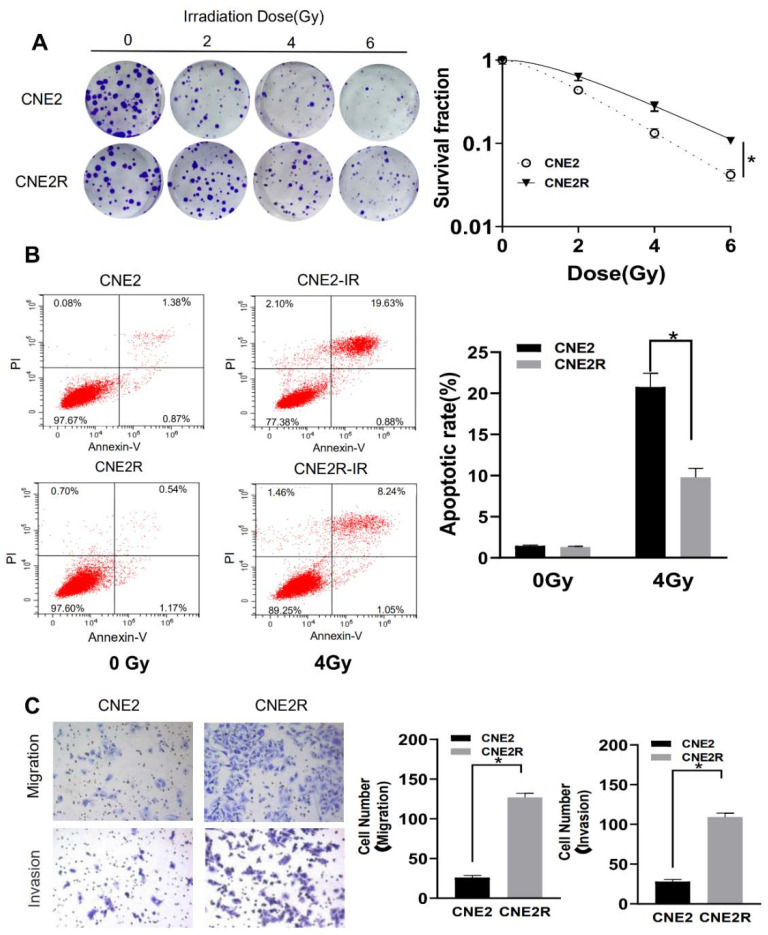
Validation of the radioresistant CNE2R cells by the measurements of colony formation, apoptosis, and cell migration. (**A**) Colony formation assay of the survival curve of CNE2R cells and its parent cell line CNE2 irradiated with different doses of γ-rays. (**B**) Flow cytometry assay of apoptosis of CNE2 and CNE2R cells irradiated with 4 Gy of γ-rays. (**C**) Migration assay and invasion assay of CNE2 and CNE2R cells. * *p* < 0.05 between indicated groups.

To identify the radioresistant phenotype, CNE2R and its parental cell line CNE2 were exposed to different doses. Colony survival assay demonstrated that CNE2R cells were much more radioresistant than CNE2 cells (Figure 1A). The plating efficiency at the doses of 0, 2, 4, and 6 Gy was 0.67, 0.29, 0.094, 0.028 for CNE2 cells and 0.51, 0.34, 0.15, 0.060, for CNE2R cells, respectively. The apoptotic rate of CNE2R cells was also significantly lower than that of CNE2 cells after 4 Gy irradiation (Figure 1B). According to the transwell assay, the migration rate and invasion ability of CNE2R cells was higher than those of CNE2 cells (Figure 1C).

### 2.2. Bioinformatics Analysis

**Figure 2:**
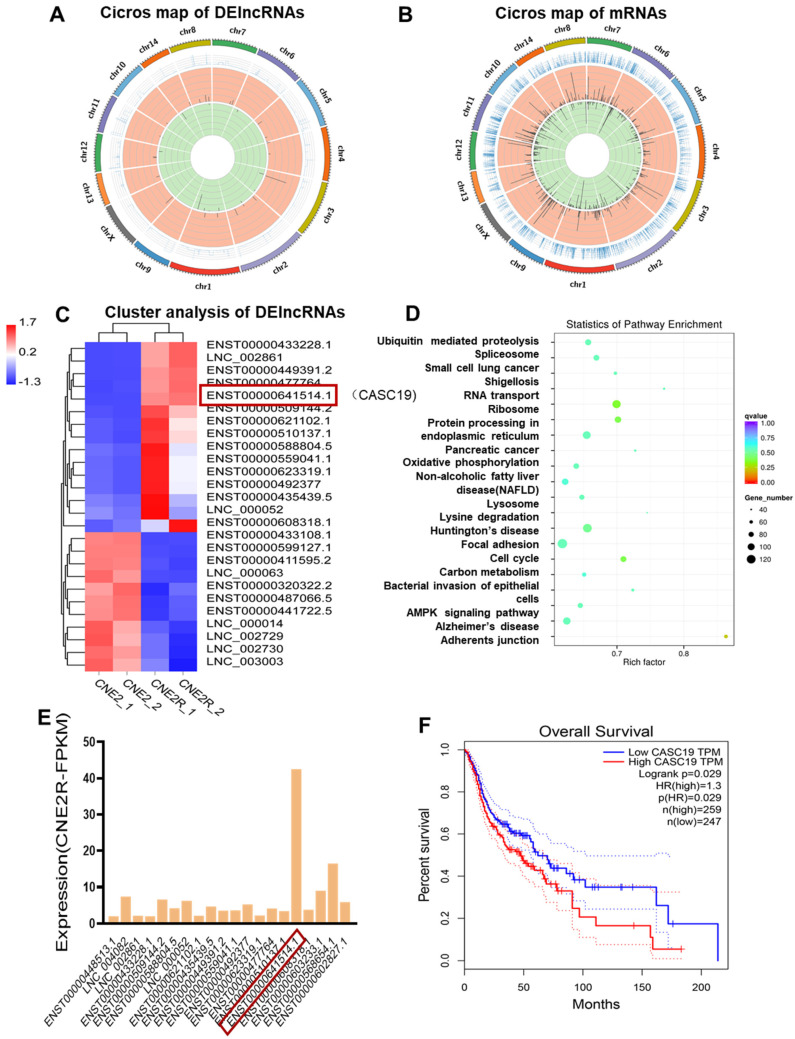
RNA-seq analysis of the paired radiosensitive and radioresistant nasopharyngeal carcinoma (NPC) cell lines CNE2 and CNE2R. (**A**,**B**) The Circos plots of total DElncRNAs and DEmRNAs between CNE2 and CNE2R cells. (**C**) The heat map plot of the distinguishable DElncRNAs with the threshold of log2 (fold changes) > 1.5 (*p* < 0.05). (**D**) KEGG pathway analysis of the DElncRNAs co-expressed mRNAs with a correlation coefficient larger than 0.995 (*p* < 0.05). (**E**) The expression abundance (FPKM, FragmentsPer Kilobase per Million) of the top 20 upregulated DElncRNAs in radioresistant cell line CNE2R. (**F**) Kaplan–Meier analysis of the relationship between the expression of CASC19 in head and neck squamous cell carcinoma (HNSC) and overall survival (OS) of the patients (data from GEPIA).

To determine the potential genes involved in the radioresistance of NPC cells, the differential lncRNA and mRNA profiles between CNE2 and CNE2R cells were explored by RNA-seq analysis, and a total of 71 DElncRNAs and 1155 DEmRNAs were identified. Among them, 47 lncRNAs and 645 mRNAs were upregulated while 24 lncRNAs and 510 mRNAs were downregulated. Moreover, the known radioresistant lncRNAs such as MALAT1 [31] and TTN-AS1 [32] were also upregulated in our RNA-seq result. NORAD, a known raised lncRNA in response to DNA damage, was also highly expressed in CNE2R cells. Others reported that lncRNAs such as UCA1, TUG1, HCG27, LINC00467, NEAT1, CYTOR, and PVT1 [23] were also increased in CNE2R cells if not significantly. Circos plots were constructed to visualize the chromosomal distribution of the DElncRNAs and DEmRNAs (Figure 2A,B). Hierarchical cluster analyses showed that the expression levels of DElncRNAs were distinguishable and varied (Figure 2C). Next, we analyzed the potential functions of the DElncRNAs by the KEGG pathway database. It was revealed that the lncRNA co-expression genes were closely related to the pathways of ubiquitin mediated proteolysis, RNA transport, oxidative phosphorylation, lysosome, cell cycle, carbon metabolism, and the AMPK signaling pathway (Figure 2D). It was found that lncRNA CASC19 was one of the most unregulated lncRNAs with the highest expression level among the top 20 DElncRNAs (Figure 2E). According to the Kaplan–Meier overall survival (OS) analysis, the prognosis of patients with a high expression of CASC19 in head and neck squamous cell carcinoma (HNSC) was significantly worse than that of HNSC patients with a low expression of CASC19 (Figure 2F) (data from GEPIA database). These results imply that lncRNA CASC19 may be involved in the development of radioresistance in NPC.

### 2.3. Knockdown of CASC19 Increased the Radiosensitivity of NPC Cells

**Figure 3:**
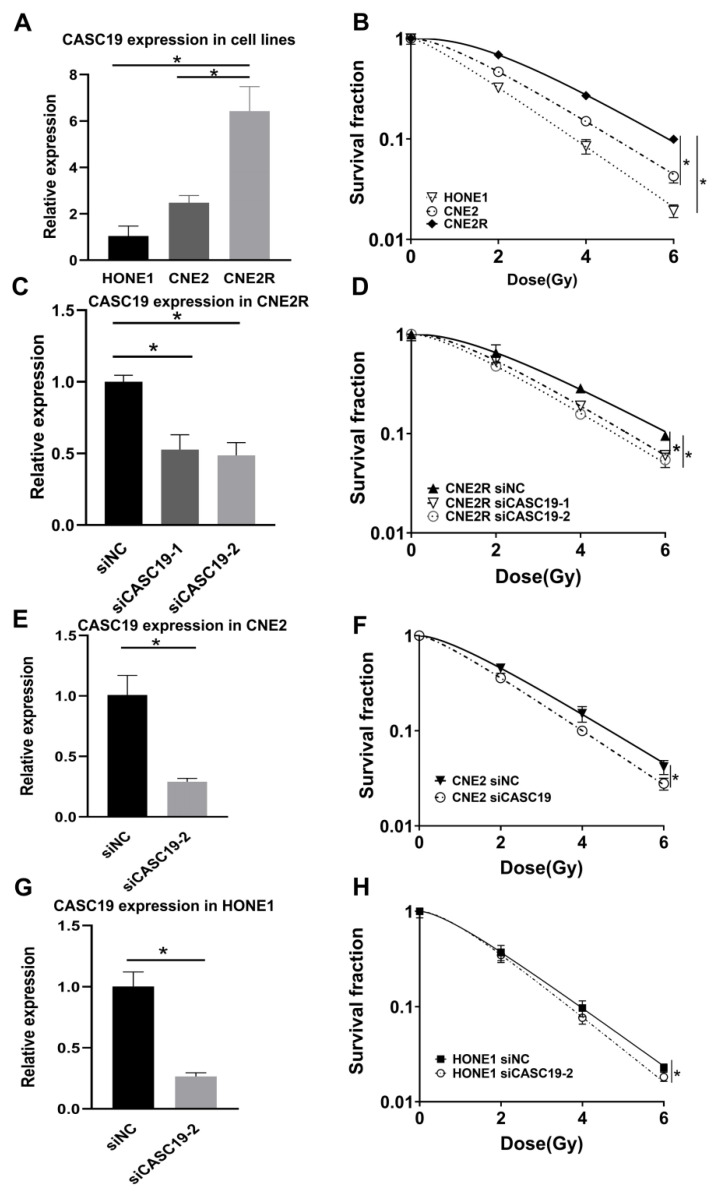
Relationship of lncRNA CASC19 expression with radiosensitivity of NPC cells. (**A**) Reverse-transcription quantitative PCR (RT-qPCR) assay of CASC19 expression in HONE1, CNE2, and CNE2R cells. (**B**) The survival factions of HONE1, CNE2, and CNE2R cells detected by clone formation assay. (**C**) The efficiency of CASC19 siRNA transfection in CNE2R cells. (**D**) Dose responses of survival factions of CNE2R cells after transfection of siCASC19-1, siCASC19-2, and control siRNA, respectively. (**E**,**G**) The influence of si-CASC19-2 on the expression of CASC19 in CNE2 cells and the survival fraction of CNE2 cells after irradiation. (**F**,**H**) The influence of si-CASC19-2 on the expression of CASC19 in HONE1 cells and the survival fraction of HONE1 cells after irradiation. * *p* < 0.05 between indicated groups.

To further assess the potential involvement of CASC19 in the radiosensitivity of NPC, we examined the expression level of CASC19 in different NPC cell lines. As a result, the expression level of CASC19 increased orderly in HONE1, CNE2, and CNE2R cells (Figure 3A), which had a positive correlation with the radioresistance of their cells (Figure 3B). The plating efficiency at the doses of 0, 2, 4, and 6 Gy was 0.45, 0.15, 0.038, and 0.009 for HONE1 cells, respectively. To further investigate the function of CASC19, two siRNAs targeting CASC19 (siCASC19-1, siCASC19-2) and a scramble control siRNA (siNC) were transferred into NPC cells. It was found that both transfection of siCASC19-1 and siCASC19-2 significantly reduced the CASC19 level in CNE2R cells in comparison with siNC (Figure 3C). These siRNA transfections also led to a remarkable reduction in the survival fraction of CNE2R cells after irradiation, where siCASC19-2 was more effective in the enhancement of radiosensitization (Figure 3D). The plating efficiency at the doses of 0, 2, 4, and 6 Gy was 0.50, 0.33, 0.14, and 0.048 for siNC-transfected CNE2R cells; 0.46, 0.25, 0.088, and 0.028 for siCASC19-1-transfected CNE2R cells; and 0.45, 0.23, 0.081, and 0.024 for siCASC19-2-transfected CNE2R cells, respectively. Thus, siCASC19-2 (hereafter referred as siCASC19) was selected to knockdown CASC19 in CNE2 and HONE1 cells. Consistently, the effective transfection of siCASC19 remarkably reduced the survival fractions of CNE2 and HONE1 cells after irradiation (Figure 3E–H). The plating efficiency at the doses of 0, 2, 4, and 6 Gy was 0.65, 0.30, 0.094, and 0.034 for siNC-transfected CNE2 cells; 0.61, 0.21, 0.061, and 0.017 for siCASC19-transfected CNE2 cells; 0.43, 0.14, 0.036, and 0.086 for siNC-transfected HONE1 cells; and 0.40, 0.014, 0.031, and 0.0073 for siCASC19-transfected HONE1 cells, respectively. However, the highest radiosensitization efficiency of siCASC19 was observed in CNE2R cells, indicating that CASC19 plays a more important role in the acquired radioresistance.

### 2.4. CASC19 Abrogation-Inhibited NPC Tumor Growth In Vivo

**Figure 4:**
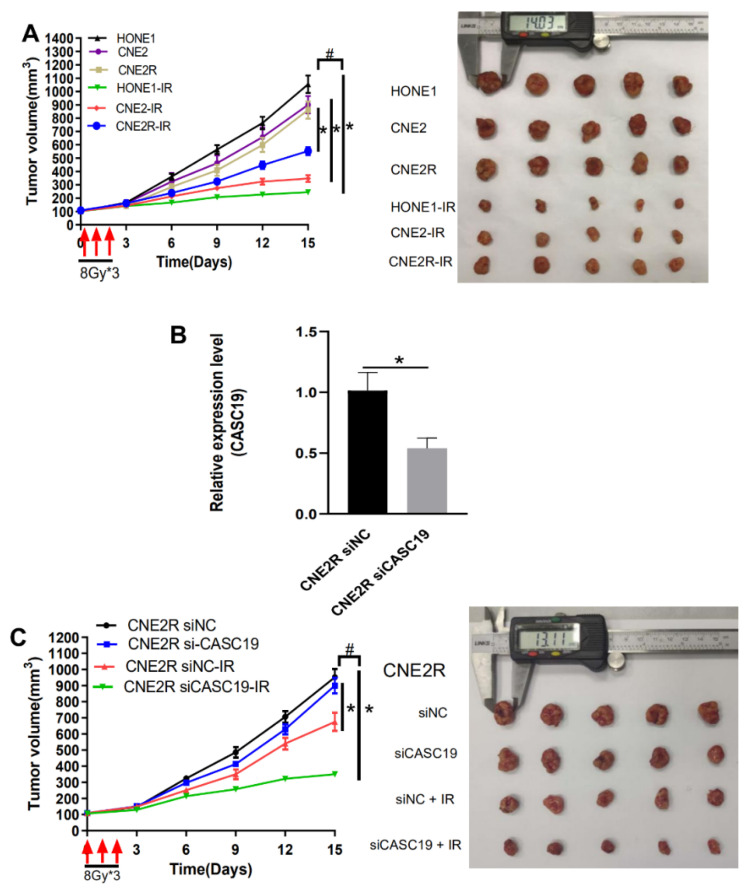
CASC19 knockdown-enhanced radiosensitivity of NPC xenograft tumors. (**A**) Tumor growth curves and representative images of HONE1, CNE2, and CNE2R xenografts in nude mice. The xenografts were irradiated with 8 Gy × 3 under a volume of about 100 mm^3^. * *p* < 0.05 between indicated groups of same cell line; # *p* < 0.05 between indicated different cell lines before and after irradiation. (**B**) RT-qPCR assay of the CASC19 expression in the CNE2R xenograft tumors that were intratumorally injected with cholesterol-modified CASC19 siRNA every 3 days from the time-point of the tumor having a volume of about 50 mm^3^ until the mice were sacrificed. (**C**) Tumor growth curves and representative image of CNE2R xenograft tumors under different treatments of siNC, siCASC19, siNC + ionizing radiation (IR), and siCASC19 + IR, respectively. * *p* < 0.05 between same cells before and after irradiation. # *p* < 0.05 between CNE2R siNC group and CNE2R siCASC19 group before and after irradiation.

To further confirm the radiosensitivity of different NPC cell lines, we irradiated the xenografts of HONE1, CNE2, and CNE2R cells in nude mice. Consistent with the in vitro experiment, the CNE2R xenograft was apparently more resistant to irradiation (8 Gy × 3) than the CNE2 xenograft, not to the mention HONE1 xenograft (Figure 4A). To determine the contribution of CASC19 in the tumor radioresistance in vivo, we administrated cholesterol-modified siCASC19 or its control into the xenograft of CNE2R cells every 3 days during tumor growth. RT-qPCR (reverse-transcription quantitative PCR) assay illustrated that the mRNA level of CASC19 in the xenograft of CNE2R cells was effectively reduced by this siCASC19 infection (Figure 4B). As expected, the intratumoral interference of CASC19 resulted in the increase in growth rate of the irradiated tumor in comparison with that of irradiated siNC cells in vivo, i.e., siCASC19 significantly reduced the radioresistance of CNE2R xenograft (Figure 4C). Accordingly, both in vitro and in vivo experiments demonstrated that lncRNA CASC19 contributed to the radioresistance of NPC.

### 2.5. Autophagy Contributed to Radioresistance of NPC Cells

**Figure 5:**
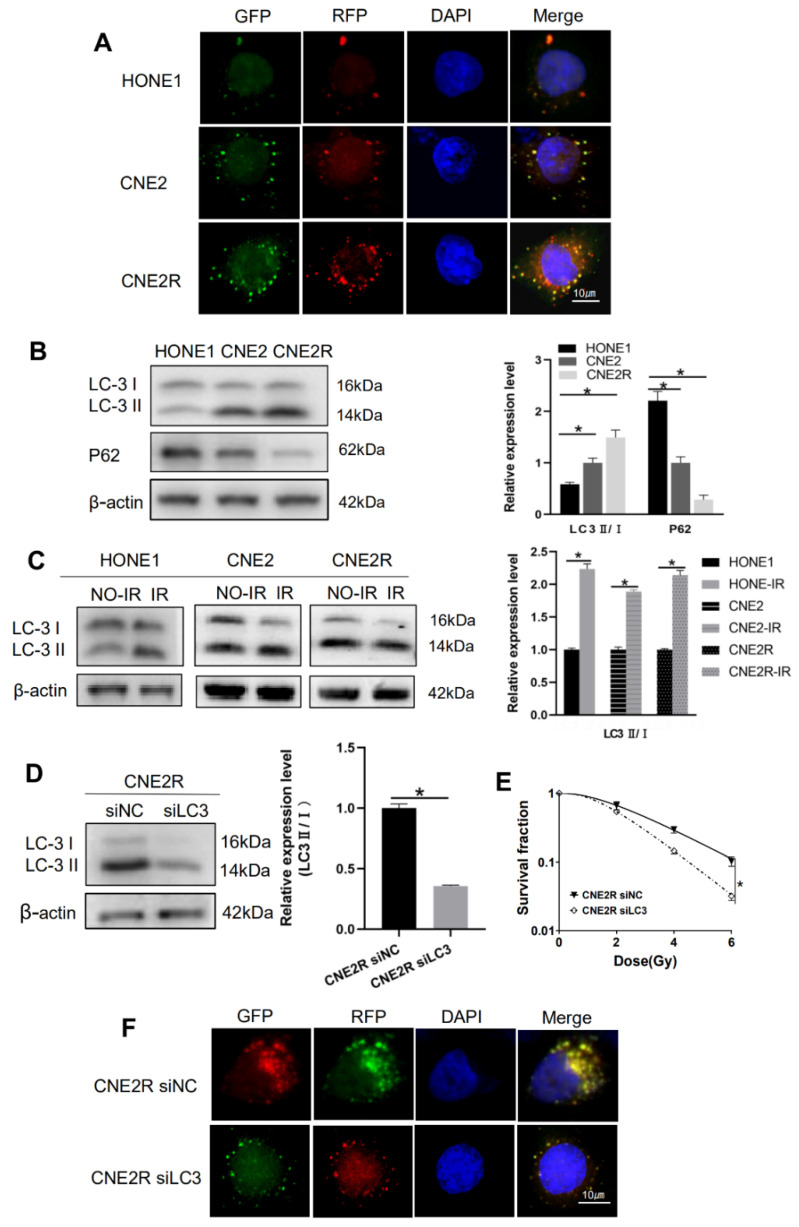
Autophagy potentiated the radioresistance of NPC cells. (**A**) Fluorescence images of HONE1, CNE2, and CNE2R cells transfected with mRFP-GFP-LC3-tagged adenovirus (×40). (**B**) Western blot assay of P62 and LC3 proteins in HONE1, CNE2, and CNE2R cells. (**C**) Western blot assay of LC3 proteins in HONE1, CNE2, and CNE2R cells after 24 h of 4 Gy radiation. (**D**) Efficiency of siLC3 transfection in CNE2R cells. (**E**) Influence of siLC3 transfection in the survival of CNE2R cells after radiation. (**F**) Fluorescence images siLC3-interfered CNE2R cells transfected with mRFP-GFP-LC3-tagged adenovirus. * *p* < 0.05 between indicated groups.

Next, we want to know the potential mechanism of the required radioresistance of NPC cells. Our previous study has shown that autophagy is an intrinsic element of radioresistance [33]. Consistently, this study revealed that, in comparison with HONE1 and CNE2, radioresistance cells CNE2R exhibited the highest intrinsic autophagy level shown as the tandem red fluorescence-green fluorescence (mRFP-GFP)-labeled-LC3 autophagosomes (Figure 5A). Western blot assay showed that the ratio of LC3II/LC3I (an autophagic marker) increased and the autophagy substrate protein p62 decreased in HONE1, CNE2, and CNE2R cells step by step (Figure 5B). Moreover, after exposure to 4 Gy X-rays, the ratio of LC3II/LC3I increased in all three cell lines (Figure 5C). When the LC3 gene was knocked-down by LC3 siRNA (Figure 5D), the survival of CNE2R cells was significantly decreased (Figure 5E) and the autophagosome formation was decreased (Figure 5F). The plating efficiency at the doses of 0, 2, 4, and 6 Gy was 0.53, 0.35, 0.16, and 0.054 for siNC-transfected CNE2R cells; and 0.51, 0.28, 0.074, and 0.016 for siLC3-transfected CNE2R cells, respectively. These results demonstrated that the increase in intrinsic autophagy level should be a reason of the acquired radioresistance of CNE2R cells.

### 2.6. Inhibition of lncRNA CASC19 Decreased Autophagy by AMPK/mTOR Pathway

**Figure 6:**
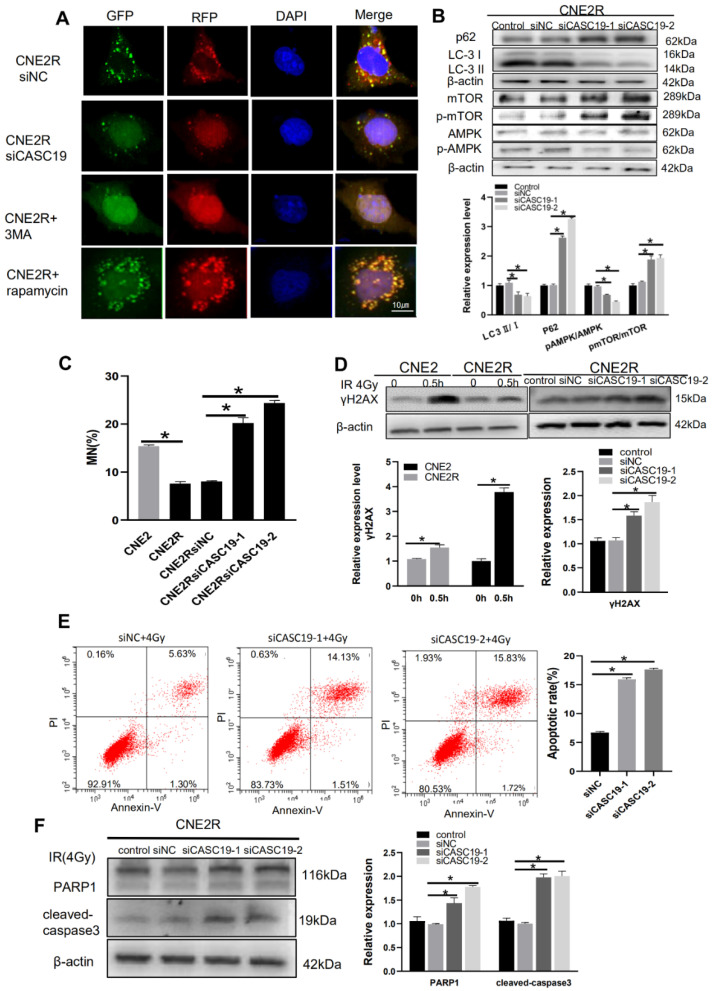
CASC19 knockdown by siRNA inhibited autophagy incidence via the AMPK/mTOR pathway and increased apoptosis in CNE2R cells. (**A**) Fluorescence images of CNE2R cells transfected with siCASC19 and mRFP-GFP-LC3-tagged adenovirus (×40). 3-MA was applied as a negative control for autophagy inhibitor and rapamycin was used as a positive control for autophagy induction. (**B**) Western blot assay of P62, LC3, p-mTOR, mTOR, p-AMPK, and AMPK proteins and their relative expression levels in CNE2R cells after transfection with siCASC19-1 and siCASC19-2. (**C**) Micronucleus formation in CNE2, CNE2R, and relevant siCASC19-transfected cells after 4 Gy irradiation. (**D**) Western blot assay of γH2AX protein in CNE2, CNE2R cells, and siCASC19-transfected CNE2R cells at 0.5 h after 4 Gy irradiation. (**E**) Flow cytometry assay of apoptosis in 4 Gy-irradiated CNE2R cells that were transfected with siCASC19 and siNC. (**F**) Western blot analysis of PARP1 and cleaved-caspase3 proteins in 4 Gy-irradiated CNE2R cells that were transfected with siCASC19 and siNC, respectively. * *p* < 0.05 between indicated groups.

To determine the relationship of CASC19 and autophagy, we transfected siCASC19 into CNE2R cells and found that this downregulation of CASC19 impaired the autophagic flux of LC3 labeled with mRFP-GFP tandem fluorescence so that the number of autophagic LC3 spots in the siCASC19-transfected cells sharply decreased to a low level (Figure 6A). In addition, 3 MA (5 mM) was used to inhibit autophagy activation as a negative control and rapamycin (0.1 µM) was applied to introduce autophagy activation as a positive control. This phenomenon was further verified by the degradation in LC3II and the increase in P62 expression in the siCASC19-transfected cells (Figure 6B).

On the other hand, Figure 2D gives a clue of the autophagy-related signaling pathway involved in radioresistance, where the AMPK pathway is one of the top enriched KEGG pathways of DElncRNAs-targeted genes. As AMPK is a well-known positive regulator of autophagy and can be acted by downregulating mTOR phosphorylation [34], we analyzed the relationship of CASC19 with the AMPK/mTOR signaling pathway. Western blot assay illustrated that, when CASC19 was downregulated by siCASC19, the expressions of p-AMPK and AMPK were obviously decreased while the expressions of p-mTOR and mTOR were increased in CNE2R cells (Figure 6B). Moreover, to corroborate the DNA damage studies, micronucleus formation was also studied. After 4 Gy irradiation, the micronucleus frequency (MN%) in CNE2R cells was obviously lower than that in CNE2 cells, and silencing CASC19 significantly increased the micronucleus frequency. Consistently, after 4 Gy irradiation, the Western blot assay showed that the γH2AX protein expression in CNE2R cells was lower than that in CNE2 cells, whereas it was increased when CNE2R cells were transfected with siCASC19 before irradiation (Figure 6C,D). These results demonstrated that the inhibition of CASC19 expression aggravated the radiation-induced DNA damage. Furthermore, CASC19-knockdown obviously elevated radiation-induced apoptosis in CNE2R cells (Figure 6E) together with the enhancement of PARP1 and cleaved caspase-3 (Figure 6F), indicating that silencing CASC19 disturbed the protective effect of autophagy on radiation-induced apoptosis. Taken together, these results demonstrate that CASC19 contributes to the radioresistance of NPC cells by promoting autophagy and inhibiting apoptosis through the AMPK/mTOR signaling pathway.

## 3. Discussion

Currently, radiotherapy is the primary therapeutic method for NPC [1]. However, an increased likelihood of recurrence and distant metastasis is still a major impediment to achieve long-term survival, and the molecular mechanism of NPC radioresistance is still unresolved [35]. After confirming the radioresistance and high migration ability of the established CNE2R cell lines, we detected the DElncRNAs between CNE2R and its parental cell line CNE2 with the bioinformatic analysis of RNA-seq, and found that lncRNA CASC19 was the most significantly upregulated gene with the highest expression abundance. Indeed, based on GEPIA database analysis, the high expression of CASC19 possesses a poor prognosis in HNSC patients.

A recent study showed that the expression of CASC19 responded with chemotherapeutic drugs in vivo and in vitro [22], but its role in tumor radiotherapy has been rarely reported. Our data revealed for the first time that CASC19 was significantly elevated in the radioresistant NPC cells. Recently, lncRNAs including PVT1, LINC00963, and HOTAIR have been reported to be associated with radioresistance in many types of cancers [7,11,36]. The characteristics of a specific expression pattern and overexpression level of lncRNAs in cancers have highlighted their potential application as the diagnosis and prognosis biomarkers of patients [37,38,39], and lncRNAs-based intervention has been an emerging area of tumor chemotherapy in combination with radiotherapy [40,41].

Moreover, it has been reported that lncRNAs may contribute to the radioresistance of cancer cells by either hampering or enhancing autophagy. For example, Zheng et al. reported that linc-RA1 knockdown could enhance radiosensitivity by activating autophagy in the glioma cell [42], while Shen et al. found that lincRNA-p21 knockdown enhanced the radiosensitivity of hypoxic tumor cells by inhibiting autophagy via the HIF-1/Akt/mTOR/P70S6K pathway in liver cancer [43].

Our KEGG analysis revealed that lysosome was one of the top enriched pathways of DElncRNAs (Figure 2C). It was reported that lysosomal damage was one of the strongest inducers of autophagy through the AMPK/mTOR axis [44]. As a conserved lysosome-mediated intracellular degradation system, autophagy is critical for the maintenance of cellular homeostasis [26] but may have functions as a double-edged sword. Autophagy can induce type-II programmed cell death through the degradation of vital components; by contrast, it may also be activated as a protector of cellular survival via adaptive response [45]. Although the role of autophagy in regulating cancer cell death or survival under different circumstances is still not clear, our results demonstrate that autophagy had a positive correlation with radioresistance.

**Figure 7:**
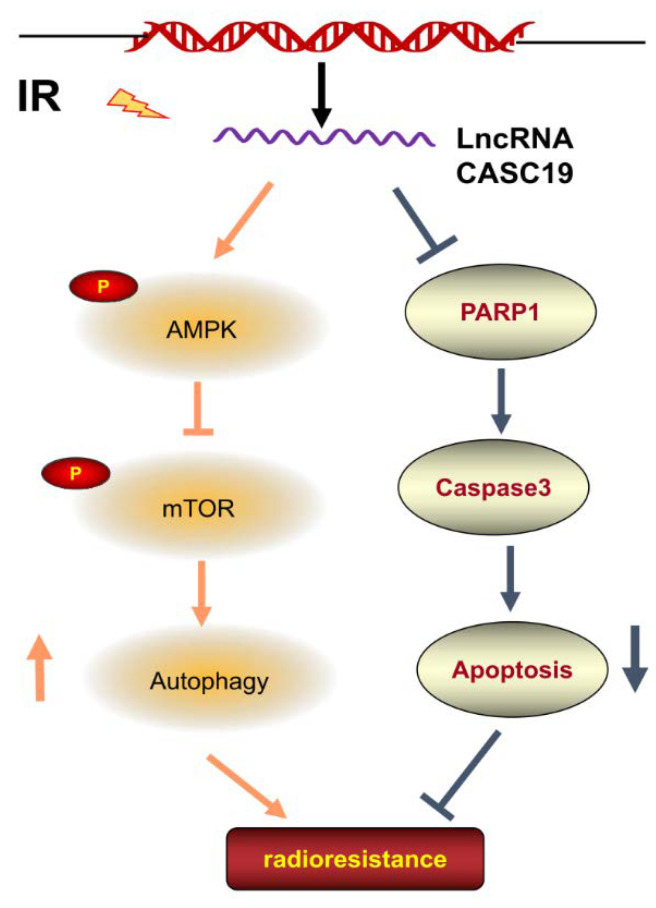
Regulation pathway of CASC19 in the radioresistance of NPC. Irradiation (IR) increased the expression of CASC19 in NPC cell lines. CASC19 contributes to autophagy via activating the AMPK/mTOR signaling pathway while inhibiting apoptosis by depressing PARP1 and cleaved-caspase3.

It has been known that AMPK, a metabolic sensor of energy balance, plays a crucial role in the regulation of energy homeostasis [46]. AMPK-triggered autophagy is mainly associated with the downregulation of mTOR [47]. Our results demonstrated that CASC19-knockdown led to the decrease in p-AMPK expression and increase in p-mTOR, indicating the contribution of the AMPK/mTOR signaling pathway in CASC19-mediated radioresistance. On the other hand, there are multiple connections between autophagy and apoptosis; the two phenomena jointly seal the fate of cells [48]. Several studies have indicated that enhancing AMPK activity may induce an anti-apoptotic effect [49,50,51]. Moreover, ionizing radiation induced micronucleus formation and activated DNA damage marker γH2AX in both radioresistant cell line CNE2R and its parental cell line CNE2 where the CNE2R appeared to have a relatively low level of DNA damage, and silencing CASC19 remarkably strengthened these radiation damages. Furthermore, serious DNA damage could induce cell apoptosis by triggering the expression of PARP1 [52], which is consistent with our result that CASC19 inhibition increased cellular radiosensitivity and apoptosis induction by increasing PARP1 and cleaved caspase-3. In conclusion, this study demonstrated that the CASC19-regulated protective autophagy through the AMPK/mTOR pathway made a major contribution to the radioresistance of NPC cells (Figure 7), suggesting that CASC19 may represent an attractive therapeutic target to improve radiotherapy against NPC.

## 4. Materials and Methods

### 4.1. Cell Culture

NPC cell lines of CNE2 and HONE1 were purchased from Shanghai Cell Bank in 2016. CNE2R, a radioresistant human NPC cell line, was previously constructed and maintained at our laboratory [30]. Briefly, CNE2 cells were irradiated with fractionated doses (2, 2, 4, 4, 4, 4, 6, 6, 6, 6, 8, 8 Gy) of γ-ray irradiation (137-Cs, Gammacell-40, MDS Nordion, Canada) at a dose rate of 0.73 Gy/min. Between two exposures, cells were cultured for nearly 7 days to recovery. After the last irradiation, the survived cells became more radioresistant than their parent cells, which was named CNE2-R cells. The cells were cultured with RPMI-1640 medium (Gibco, Hangzhou, China) supplied with 10% fetal bovine serum (Gibco Invitrogen, Grand Island, NY, United States), 100 U/mL penicillin, and 100 mg/mL streptomycin, and maintained at 37 °C in a humidified atmosphere of 5% CO_2_ and 95% O_2_.

### 4.2. Colony Formation Assay

The survival fractions of irradiated CNE2 and CNE2R cells were determined by cell colony-formation assay. After mock irradiation (0 Gy) or irradiation with different doses (2, 4, 6 Gy) of γ-rays, the cells were incubated for about 10 days until colony appearance. The colonies were fixed with methanol for 20 min and stained with 0.1% crystal violet for 30 min. Colonies with more than 50 cells were counted. The cell survival curves were analyzed by the single-hit multitarget model using GraphPad Prism 8.0 software.

### 4.3. Apoptosis Analysis

Cells were irradiated with 4 Gy γ-rays, and after 48 h, the cells were washed with cold PBS triply and then collected in 50 µL of suspension buffer containing 2.5 µL Annexin V-FITC and 2.5 µL PI (TransGen Biotech, Beijing, China). Then, 200 µL buffer was added to the solution after incubation for about 15 min in the dark at room temperature; then, cell apoptosis was detected using flow cytometry (Beckman CytoFLEX, CA, USA).

### 4.4. Transwell Assay

Transwell inserts with 8.0 µm pores on the bottom (Corning Incorporated, Corning, NY, USA) were employed to examine cell migration and invasion ability. The difference between cell migration and invasion assay was whether the bottom of the insert chamber was pre-coated with or without matrigel. The cells were seeded into the insert dishes with serum-free medium while the lower chambers were supplemented with medium containing 10% FBS. After 24 h of incubation, the cells migrated to the lower surface of the insert dish were fixed (10% methanol, 15 min, 37 °C), stained (0.1% crystal violet, 10 min, 37 °C), and imaged (Olympus, Tokyo, Japan).

### 4.5. RNA Isolation, Library Preparation, and Sequencing Analysis

Total cellular RNA was extracted using TRIzol reagent (Invitrogen, San Diego, CA, USA) according to the manufacturer’s instructions. In addition, 20 ng of purity and integrity RNA per sample was used for sequencing analysis. RNA-sequencing (RNA-seq) libraries were generated using the rRNA-depleted RNA by NEBNext^®^ Ultra™ Directional RNA Library Prep Kit for Illumina^®^ (NEB, Ipswich, MA, USA) following manufacturer’s recommendations, and the library quality was assessed on the Agilent Bioanalyzer 2100 system. After cluster generation, the libraries were sequenced on an Illumina Hiseq 2500 platform. The Ballgown method was used to compare the expression of lncRNAs and mRNAs between different cell samples (CNE2-1, CNE2-2 vs. CNE2R-1, CNE2R-2). Transcripts with *p*-value < 0.05 were assigned as differentially expressed lncRNAs and differentially expressed mRNAs (DEmRNAs). All sequencing programs and analyses were performed by Novogene Company (Beijing, China).

### 4.6. lncRNA Co-Expression Analysis and lncRNA Target Prediction

To understand how lncRNAs influence the protein-coding genes, functional annotations of their co-expressed mRNAs are used to predict functions of lncRNAs. The expressed correlation between differentially expressed lncRNAs (DElncRNAs) and coding genes were calculated with R function cor.test (a test for association/correlation between paired samples) to compute Pearson’s correlation coefficient. The absolute value of Pearson’s correlation coefficient was ≥0.995 (*p* < 0.05). The KEGG pathway database was applied to analyze the potential functions of these target genes using KOBAS software [53].

### 4.7. Western Blotting

Total cellular proteins were extracted with RIPA buffer (Beyotime Biotechnology, Shanghai, China), separated by SDS-PAGE, and transferred to a PVDF membrane (Millipore, Bedford, MA, USA). The membrane was blocked with 5% nonfat milk in Tris buffer saline/Tween 0.05% (TBST) for 2 h and incubated with primary antibodies overnight at 4 °C. After washing with TBST triply, the membrane was incubated with HRP (horseradish peroxidase)-conjugated secondary antibodies for visualization.

### 4.8. RNA Extraction and Quantitative RT-qPCR Assay

TRIzol reagent was used to extract total RNA from HONE1, CNE2, and CNE2R cells and fresh tissues for RT-qPCR assay. Total RNA (1 μg) was reversely transcribed into cDNA using the primeScript RT reagent kit with gDNA Eraser (Takara Biotechnology, Co., Ltd., Dalian, China). qRT-PCR was applied with Ultra SYBR Mixture (Low ROX) (CoWin Biosciences, Beijing, China) in 25 µL reaction reagents using the MX3000P platform according to the manufacturer’s protocol. For the CASC19 gene, the forward primer was TTT AGC CTG CAT AGG ACC CTC and the reverse primer was GTC TGG TCA AAT TAC AAT CAG TTGG. For the β-actin gene, the forward primer was CAT GTA CGT TGC TAT CCA GGC and the reverse primer was CTC CTT AAT GTC ACG CAC GAT. The PCR amplification procedure was performed by 40 cycles with pre-denaturation at 95 °C for 10 min, denaturation at 95 °C for 15 s, and annealing and extension at 60 °C for 1 min.

### 4.9. RNA Interference and Drug Treatment

HONE1, CNE2, and CNE2R cells were transferred with CASC19 siRNA (siCASC19) or LC3 siRNA (siLC3) using riboFECT CP Transfection Agent (RiboBio, Guangzhou, China) according to the manufacture’s protocol. The sequences of siRNAs are listed as follows: siCASC19-1 (GCT CAG CAT TTG CCA TACT), siCASC19-2 (CCT TAG AAT TGG AGT GCCT), siLC3 (GAG UGA GAA AGA UGA AGA UTT). The negative control of siRNA has a random sequence. Transduction efficiency was consistently between 90 and 95%. 3-MA (MCE, HY-19312) and rapamycin (MCE, AY-22989) were used as the autophagy inhibitor and autophagy inducer, respectively. CNE2R cells were treated with 5 mM 3-MA or 0.1 µM rapamycin for 6 h.

### 4.10. Micronucleus (MN) Assay

Cells were seeded in the 6-well plate and allowed to adhere overnight prior to transfected treatment. After 24 h transfection, the cells were given 4 Gy irradiation and subsequently incubated for 24 h in RPMI-1640 (with 10% FCS) containing cytochalasin B (1.0 µg/mL) to block cytokinesis. Then, the cells were washed and fixed in fixatives (methanol:acetic acid = 85:15) for 15 min after hypotonic treatment with 0.075 mol/L KCL for 1 min and stained with 2.5 μg/mL AO (acridine orange). Micronuclei were scored using a fluorescence microscope.

### 4.11. Analysis of Autophagic Flux

Autophagy was examined by analyzing the formation of fluorescent puncta of autophagosomes in the cells transfected with mRFP-GFP-LC3-tagged adenovirus (Hanbio Biotechnology Co., Shanghai, China). Briefly, approximately 3 × 10^5^ cells/well were incubated in a 24-well plate overnight. After 2 h of transfection with the adenovirus (diluted in serum-free medium), the cells were cultured in fresh medium for 48 h. Then, the cells were washed triply with pre-cold PBS (PH 7.4), fixed, and observed under a high-content imaging system (Image Xpress Micro 4, Molecular Devices, San Francisco, CA, USA). In the photo image, yellow puncta represent the merge of GFP and RFP signals, while red (RFP signal alone) puncta indicate the late autolysosomes.

### 4.12. Tumor Radiosensitivity Assay

Nude male mice (BALB/C-nu/nu) of 4 weeks-old were purchased from the Laboratory Animal Center of SLAC (Shanghai, China) and housed with free access to distilled food and water in IVCs (individually ventilated cages) at 24 °C on a 12 h light-dark cycle. Then, 30 mice were equally divided into six groups for the administration of HONE1, CNE2, and CNE2R cells where half of them were applied for irradiation. A total of 4 × 10^6^ cells were subcutaneously injected into the right flanks of 5 week old nude mice. When the average volume arched to approximately 100 mm^3^, the tumors were given a local radiation (8 Gy) for 3 consecutive days. After 15 days of the last irradiation, the mice were sacrificed and the tumors were harvested.

Another 20 mice were applied to evaluate the therapeutic effect of interference with siCASC19 in vivo. Briefly, CNE2R cells (4 × 10^6^) were subcutaneously injected into the right flanks of mice. When the xenograft volumes approached approximately 50 mm^3^, cholesterol-modified CASC19 siRNA or its control (RiboBio, Guangzhou, China) was administrated intratumorally every 3 days until the mice were sacrificed. This protocol has been proven to be effective in reducing gene expression in vivo [54]. When the tumors reached an average volume of approximately 100 mm^3^, they were exposed to local radiation of 8 Gy × 3. After irradiation, tumor size was measured with a caliper every 3 days and calculated by using the modified ellipse formula (volume = length × width × height × π/6). All animal experiments were approved by the Animal Welfare and Ethics Committee of Fudan University (20171304A215, 9 February 2017)

### 4.13. Statistical Analysis

All experiments were repeated at least three times and the data are presented as the mean ± SD. The difference between indicated groups was evaluated by Student’s t-test or one-way analysis of variance (ANOVA) using SPSS 19.0 software (SPSS, Chicago, IL, USA). RNA-seq was analyzed by specific R packages. *p* < 0.05 was considered statistically significant.

## Data Availability

The data presented in this study are available on request from the corresponding author upon reasonable request.
